# Associations between learning environment variables and students’ approaches to studying: a cross-sectional study

**DOI:** 10.1186/s12909-020-02033-4

**Published:** 2020-04-20

**Authors:** Gry Mørk, Trine A. Magne, Tove Carstensen, Linda Stigen, Lene A. Åsli, Astrid Gramstad, Susanne G. Johnson, Tore Bonsaksen

**Affiliations:** 1 Faculty of Health Studies, VID Specialized University, Sandnes, Norway; 2 Department of Health Sciences, Norwegian University of Science and Technology (NTNU), Trondheim, Norway; 3 Department of Health Sciences, Norwegian University of Science and Technology (NTNU), Gjøvik, Norway; 4 Department of Health and Care Sciences, Faculty of Health Sciences, UiT The Arctic University of Norway, Tromsø, Norway; 5 Centre for Care Research, North, Tromsø, Norway; 6 Department of Occupational Therapy, Faculty of health and function, Western Norway University of Applied Sciences, Bergen, Norway; 7 Department of Occupational Therapy, Prosthetics and Orthotics, Faculty of Health Sciences, Oslo Metropolitan University, Oslo, Norway

**Keywords:** Approaches to studying, Higher education, Learning environment, Occupational therapy, Students

## Abstract

**Background:**

Aspects of the learning environment may be related to students` approaches to studying, but few studies have investigated these relationships in the context of occupational therapy education.

**Objective:**

To examine associations between occupational therapy students’ perceptions of the learning environment and their approaches to studying.

**Method:**

One hundred eighty-seven first-year occupational therapy students in Norway (response rate 61.3%) participated in this study. Aside from sociodemographic information, the students completed the *Course Experience Questionnaire* and the *Approaches and Study Skills Inventory for Students*. Associations between learning environment variables and study approaches were investigated with hierarchical linear regression analyses.

**Results:**

Higher scores on *Generic skills* were associated with higher scores on the deep and strategic approach scales (*β* ranging 0.18–0.51), while lower scores were associated with higher surface approach scale scores (*β* = − 0.24). Lower scores on *Clear goals and standards* and *Appropriate workload* were associated with higher surface approach scores (*β* ranging − 0.16 - -0.42).

**Conclusion:**

By improving aspects of the learning environment, there may be a potential for influencing occupational therapy students’ approaches to studying. Based on this study, emphasizing how generic skills developed in the study program may become useful in practising a profession, ensuring clarity of goals and standards, and maintaining an appropriate workload on students appear to be important.

## Introduction

Learning outcomes are the intended competency products of a students’ learning process throughout an education program. They are formulated to reflect different levels of knowledge, skills and general competency embedded in the three levels of higher education [1]. In Norway, the occupational therapy education program is a three-year program at the bachelor’s degree level. According to the Norwegian National Qualifications Framework, general learning outcomes for education programs at the bachelor’s degree level include, for example, having “broad knowledge of core topics, theories, problems, processes, tools and methods within the subject area” ([1], p. 23). According to the Structure of Observed Learning Outcomes (SOLO) taxonomy [2], this learning outcome reflects the multi-structural level, as it requires the student to have overview of different aspects related to a given topic.

Another example of a general learning outcome for bachelor-level education is to be able to “apply professional knowledge and relevant results from research and development onto practical and theoretical problems, and make justified decisions” ([1], p. 23). This, and similar learning outcomes, reflects a higher level of learning as they require students to integrate different aspects of knowledge into a coherent, integrated whole (relational level), and to generalize and apply that knowledge onto new problems (extended abstract level) [2]. Illustrated by the above examples, we would argue that many of the general learning outcomes for bachelor-level education in Norway, such as occupational therapy education, reflect learning at the multi-structural level or higher. Currently established views on learning tend to emphasize the students’ own behaviors and actions to be the main driving forces of learning [3–5]. Thus, an important question becomes what types of student behaviors would assist students in achieving higher-level learning outcomes.

Study behaviors, when generalized, are often denoted ‘approaches to studying’. Building on a large body of theory and research, and heavily influenced by Noel Entwistle and co-workers, three main approaches to studying have been identified to characterize students’ general orientation towards studying in academic settings: *the deep, surface and strategic approaches* [4–8]. The deep approach reflects the students’ purpose to increase his or her understanding of the topic. Using the deep approach to studying, the student attempts to connect and distinguish between the different ideas introduced in the study materials. Using the surface approach to studying, by contrast, the student does not truly engage with the studies, but attempts to avoid failing exams while using only the minimum of effort required. The third approach to studying, the strategic approach, is oriented towards achievement. Using this approach, the student aims at achieving good grades and organizes his or her study efforts accordingly.

Empirical research in the fields of psychology and health care education has found the use of deep and strategic study approaches to be related to better clinical and academic performance outcomes, whereas surface approach behaviors have been related to poorer outcomes [9–16]. A largely similar pattern of associations has been found in studies of occupational therapy students [17]. In view of the existing research, the deep and strategic approaches to studying appear to be better suited to achieve the higher-level learning outcomes predominant at the higher education level, when compared with the surface approach.

In several studies, researchers have found associations between learning environment factors and study approaches [18–22]. Further, studies have suggested that specific pedagogical approaches that effectively modify the learning environment can encourage a deep approach to studying [23–25]. Generic skills, such as analytical-, communication-, teamwork- and problem-solving skills, are seen as learning outcomes required for working life as well as for studying [18, 20, 22]. Nonetheless, the line of research examining students’ approaches to studying in context of the learning environment is relatively new. It has rarely been pursued with occupational therapy students and, to date, no similar studies have been conducted in Norway. Thus, there is a need to explore empirically the possible associations between learning environment perceptions and students’ approaches to studying in occupational therapy students. Increased knowledge about the environments associated with students’ approaches to studying may enable occupational therapy educators to modify relevant aspects of the learning environment to encourage their students’ use of productive study behaviors, which in turn may increase their learning outcomes.

### Study aim

The aim of the study was to examine associations between occupational therapy students’ perceptions of the learning environment and their approaches to studying, while adjusting for sociodemographic characteristics.

## Methods

### Design and study context

The study is part of a longitudinal study of occupational therapy students’ perceptions of the learning environment and approaches to studying. In the current study, cross-sectional data from students enrolled in the first year of the study program were used.

### Participants

First year occupational therapy students at six higher education institutions in Norway were approached for possible inclusion in the study.

### Measurement

#### Sociodemographic variables

Age (in years) and time spent on independent studying (average hours during a typical week) were registered as continuous variables. Gender (male = 0, female = 1), having prior experience from higher education (no = 0, yes = 1) and having occupational therapy as the highest prioritized line of education at the time of enrolment (no = 0, yes = 1) were registered as categorical variables.

#### The learning environment

The original *Course Experience Questionnaire* (CEQ, [26]) consists of 30 items distributed onto five scales: clear goals and standards, emphasis on independence, good teaching, appropriate workload, and appropriate assessment. In addition to the 30 items, one item assesses the students’ general satisfaction with the course. Later, a 37 items ‘long version’ of the CEQ has been established [20, 27, 28], including a sixth scale concerned with generic skills, and the validated Norwegian translation of this version [29] was used in the present study. Higher scores on the scales indicate that the respondent perceives the course to have (i) clearly established and disseminated goals; (ii) high levels of student autonomy and independence; (iii) teaching that engages and involves the students; (iv) a workload that is not too high; (v) assessment forms that promote and support learning; and the course is felt to (vi) support the transfer of knowledge and skills to the relevant work context. In the current study, internal consistency measures were 0.73 (clear goals and standards), 0.63 (emphasis on independence), 0.70 (good teaching), 0.69 (appropriate workload), 0.45 (appropriate assessment), and 0.83 (generic skills). In view of the preliminary internal consistency results, the ‘appropriate assessment’ scale was removed from the subsequent analyses [30]. Table 1 displays example items from each of the employed CEQ scales.

**Table 1:** Scales and example items from the *Course Experience Questionnaire*

Scales	Items
Clear goals and standards	The aims and objectives of this course are not made very clear^a^
Student autonomy	Students have a great deal of choice over how they are going to learn in this course
Good teaching	The staff make a real effort to understand difficulties students may be having with their work
Appropriate workload	The sheer volume of work to be got through in this course means you can’t comprehend it all thoroughly^a^
Generic skills	This course has helped develop my ability to work as a team member

The scale ‘Appropriate assessment’ was excluded from the current study. ^a^The item has reversed coding

#### Approaches to studying

Study approaches were measured with the *Approaches and Study Skills Inventory for Students* (ASSIST, [31]), and the students used a previously validated Norwegian translation of the instrument [32]. The ASSIST consists of 52 statements to which the respondent is asked to rate his or her level of agreement (1 = disagree, 2 = disagree somewhat, 3 = unsure, 4 = agree somewhat, 5 = agree). The instrument has a three-factor structure, a structure recently replicated in a cross-cultural study of undergraduate occupational therapy students [33]. The items are organized accordingly into three main scales (the *deep, strategic,* and *surface* approaches to studying). Scale scores are calculated by adding the scores on the relevant items. In this study, internal consistency estimates (Cronbach’s α) for the study approach scales were 0.71 (deep approach), 0.84 (strategic approach), and 0.76 (surface approach). Table 2 displays example items from each of the three ASSIST scales.

**Table 2:** Scales and example items from the *Approaches and Study Skills Inventory for Students*

Scales	Items
Deep approach	I try to relate ideas I come across to those in other topics or other courses whenever possible. When I have finished a piece of work, I check it through to see if it really meets the requirements.
Strategic approach	I think I’m quite systematic and organised when it comes to revising for exams. I look carefully at tutors’ comments on course work to see how to get higher marks next time.
Surface approach	I’m not really interested in this course, but I have to take it for other reasons. I like to be told precisely what to do in essays or other assignments.

### Data analysis

The sample was described with descriptive statistics; i.e., means and standard deviations for continuous variables and frequencies and percentages for categorical variables. Comparisons between men and women were performed using Chi-square tests (categorical variables) and independent *t*-tests (continuous variables). Three subsequent hierarchical linear regression analyses were performed, using the deep, strategic and surface approach scales as outcome variables. For each of the regression analyses, independent variables were included in two subsequent blocks: (i) the sociodemographic factors: age, gender, time spent on independent study, educational priority, prior higher education; and (ii) the learning environment factors: clear goals and standards, student autonomy, good teaching, appropriate workload, and generic skills. Effect sizes were reported as standardized *β* coefficients, and statistical significance was set at *p* < 0.05.

### Research ethics

Approval for collecting, storing and utilizing the de-identified data was granted on October 12, 2017 by the Norwegian Center for Research Data (project no. 55875).

## Results

### Response rates

From the six education programs, 305 students were eligible participants, and of these 187 students (response rate 61.3%) participated. For each of the institutions, the response rates were 24/76 = 31.6% in Oslo, 56/77 = 72.7% in Trondheim, 19/39 = 48.7% in Gjøvik, 31/47 = 66.0% in Sandnes, 24/24 = 100.0% in Tromsø, and 33/42 = 78.6% in Bergen.

### Sample characteristics

Table 3 displays background characteristics, perceptions of the learning environment, and approaches to studying in the sample and for men and women separately. One participant did not indicate sex. Men (*n* = 37, *M* = 24.5 years, *SD* = 4.6 years) were older than women (*n* = 149, *M* = 22.5 years, *SD* = 4.3 years, *p* = 0.01), and compared to women (37.6%), a larger proportion of men (59.5%) had higher education experience prior to enrolment into the occupational therapy program (*p* = 0.02). Women had significantly higher scores on strategic approach to studying, compared to men (*M* = 72.9, *SD* = 10.4 vs. *M* = 68.8, *SD* = 9.4, *p* = 0.03). Otherwise, no systematic gender differences occurred with regard to the included variables.

**Table 3:** Sociodemographic characteristics, perceptions of the learning environment, and approaches to studying in the sample and among men and women

Variables		Total	Men (*n* = 37)	Women (*n* = 149)
*Sociodemographic variables*	*n*	*M (SD)*	*M (SD)*	*M (SD)*
Age	185	22.9 (4.6)	24.5 (5.5)	22.5 (4.3)
Time on independent study	182	9.3 (7.0)	9.6 (7.9)	9.3 (6.8)
		*n (%)*	*n (%)*	*n (%)*
Priority line of study	186	117 (62.9)	24 (64.9)	93 (62.4)
Prior higher education	186	78 (41.9)	22 (59.5)	56 (37.6)
*Learning environment*		*M (SD)*	*M (SD)*	*M (SD)*
Clear goals and standards	185	16.6 (3.9)	17.0 (3.9)	16.5 (3.9)
Student autonomy	186	18.6 (4.2)	18.4 (4.6)	18.7 (4.1)
Good teaching	185	27.2 (6.2)	26.3 (5.6)	27.4 (6.4)
Appropriate workload	186	15.2 (3.7)	15.7 (3.5)	15.0 (3.8)
Generic skills	186	22.9 (4.1)	23.1 (3.4)	22.8 (4.3)
*Approaches to studying*				
Deep approach	186	56.6 (8.6)	58.9 (11.2)	56.0 (7.8)
Strategic approach	186	72.1 (10.3)	68.8 (9.4)	72.9 (10.4)
Surface approach	186	47.3 (9.2)	45.4 (9.8)	47.8 (9.1)

When *n* < 187 the valid percent is stated. The variable ‘Time on independent study’ is average number of hours spent on independent studying during a typical week

### Adjusted associations with the study approach scales

Table 4 displays the results from the regression analysis, using the students’ scores on the deep, strategic and surface study approaches as dependent variables in three subsequent analyses. Higher age (*β* = 0.13, *p* < 0.05), having prior higher education (*β* = 0.16, *p* < 0.05) and having a stronger sense of developing generic skills during the study program (*β* = 0.51, *p* < 0.001) were directly associated with higher deep approach scores. The full model accounted for 30.7% of the variance in deep approach scores.

**Table 4:** Hierarchical linear regression analyses showing adjusted associations with scores on the study approach scales

Independent variables	Deep approach		Strategic approach		Surface approach	
*Sociodemographic variables*	*β*	*p*	*β*	*p*	*β*	*p*
Age	0.13	< 0.05	0.01	0.90	−0.12	0.07
Sex	−0.07	0.32	0.18	< 0.05	0.02	0.73
Time on independent study	0.10	0.13	0.23	< 0.01	− 0.01	0.82
Priority line of study	−0.02	0.81	0.07	0.34	−0.18	< 0.01
Prior higher education	0.16	< 0.05	0.01	0.86	−0.02	0.79
**Explained variance**	**6.2%**	**< 0.05**	**7.7%**	**< 0.05**	**7.5%**	**< 0.05**
*Learning environment*						
Clear goals and standards	−0.13	0.10	0.14	0.09	−0.16	< 0.05
Student autonomy	0.00	> 0.99	0.12	0.18	0.15	0.06
Good teaching	0.08	0.39	−0.01	0.92	0.02	0.85
Appropriate workload	−0.10	0.17	0.07	0.39	−0.42	< 0.001
Generic skills	0.51	< 0.001	0.18	< 0.05	−0.24	< 0.01
**R**^**2**^**change**	**24.5%**	**< 0.001**	**13.2%**	**< 0.001**	**29.4%**	**< 0.001**
**Explained variance**	**30.7%**	**< 0.001**	**20.9%**	**< 0.001**	**36.9%**	**< 0.001**

Table content is standardized beta (*β*) values with corresponding significance (*p*) levels. The variable ‘Time on independent study’ is average number of hours spent on independent studying during a typical week. Men were coded ‘0’ and women ‘1’, i.e., women had higher scores on the strategic approach to studying

Being female (*β* = 0.18, *p* < 0.01), spending more time on independent study (*β* = 0.23, *p* < 0.01) and having a stronger sense of developing generic skills during the study program (*β* = 0.18, *p* < 0.05) were directly associated with higher strategic approach scores. The full model accounted for 20.9% of the variance in strategic approach scores.

Students who did not have occupational therapy as their first priority line of study at the time of enrolment had higher surface approach scores (*β* = − 0.18, *p* < 0.01), compared to their counterparts. Higher scores on surface approach were also observed for students who perceived the workload to be too high (*β* = − 0.42, *p* < 0.001), the goals and standards to be unclear (*β* = − 0.16, *p* < 0.05), and to a lesser extent perceived that they developed generic skills during the study program (*β* = − 0.24, *p* < 0.01). The full model accounted for 36.9% of the variance in surface approach scores.

## Discussion

The aim of the study was to examine associations between occupational therapy students’ perceptions of the learning environment and their approaches to studying while adjusting for sociodemographic factors. The generic skills scale was associated with all three study approaches, while the clear goals and standards and appropriate workload scales were associated with the students’ scores on the surface approach. All significant associations were in the direction predicted from theory.

### Relationships between generic skills and approaches to studying

Compared to their counterparts, students with higher scores on generic skills were more inclined to have higher scores on the strategic approach scale, and – in particular – the deep approach scale. These findings are in line with the findings reported by Tuononen and co-workers [22]. Conversely, lower scores on generic skills were associated with higher surface approach scale scores. According to Entwistle ([4], p. 70), students with a deep approach to learning “*integrate the whole with its purpose, showing an intention to impose meaning on the content in relation to the perceived nature of the task, trying to “stand back” from the task, thinking about the underlying structure and seeing it in a wider perspective”*. This description of the deep learner strongly mirror the description of the generic skills scale, emphasizing analytic and problem-solving skills transferable to new situations [26, 29]. Thus, a degree of conceptual overlap may explain the strong association between the two scales. In a similar vein, Beccaria and co-workers [34] found a strong relationship between meta-cognitive awareness and the deep approach to learning. Meta-cognitive awareness was also related to time management, goal setting, and self-reflecting as a group member, which align with the concept of generic skills as used in the current study.

The associations between generic skills and the study approach measures indicate that students using deep and/or strategic study approaches employ a fuller range of desired learning activities, including reflection, theorizing and application. According to Biggs’ SOLO taxonomy [2, 3], these learning activities reflect learning at the relational and extended abstract levels. However, for students using a surface approach, there is a shortfall. These students tend to handle all tasks, regardless of their complexity, with low-level learning processes (such as memorizing and recalling) without connecting the meanings embedded in the concepts they try to recall [2]. As a result, learning based on a surface approach tends to be a recollection of terms, rather than an understanding of interconnected concepts. Conversely, a learning environment that fails to facilitate reflection, analysis and problem-solving (as reflected in low scores on generic skills) may negativity affect the student’s motivation and sense of meaning, potentially increasing surface approach learning behaviors. In such cases, students tend to treat tasks as something external, avoid extracting a deeper meaning from what they study, and tend to focus on elements, rather than the whole [4].

The consistent associations between generic skills and approaches to studying, as detected in this study, indicate that one possible way of changing students’ approaches to studying can be to assist them in transferring classroom-based knowledge and skills onto new situations. Variation in teaching methods and pedagogical practices are required for the learning of generic skills [35], as are students’ use of active learning strategies [36]. At the same time, oppositely directed associations are equally viable, as using deep/strategic study approaches may make students more attuned towards the possible practical applications of their learning [29].

### Relationships between appropriate workload and clear goals, and approaches to studying

We found that lower scores on ‘appropriate workload’ were associated with higher surface approach scores. According to Lizzio and co-workers [20], this is one of the most consistent findings in the field. For example, Diseth [18] found that perceived heavy workload was related to surface approach studying, whereas students who were more satisfied with the level of workload had higher levels of deep and/or strategic approach behaviors. Diseth’s results also showed that “workload” was the only learning environment variable which correlated with examination grades – perceiving the workload to be too heavy had an independent, direct effect on lower examination grades. Conversely, students who have adopted a surface approach to studying with a preference for simple and uncomplicated tasks may have low self-efficacy [37], and may therefore be inclined to perceive the workload as too heavy. For the students in the current sample, mandatory learning activities such as group work and practical skills training, constitute a substantial part. For students with a surface approach to studying, indicating having *little personal engagement in the task or a feeling that the task is an unwelcome imposition by authority* ([4], p. 72), such learning activities may therefore represent tiresome additions to the workload. This may explain the association between higher surface approach scores and perceiving the workload as to heavy.

It may be serviceable to conceive students’ approach to studying as potentially influenced by their learning environment. A heavy workload might cause motivation to decline and instigate a fear of failure, which in turn may lead to surface approach study behaviors. Students who fear failure may feel overwhelmed with the amount of study materials and can start panicking if they feel behind with the work. As demonstrated by Bonsaksen and co-workers [17], having higher scores on the ‘fear of failure’ dimension of the surface approach was associated with poorer academic performance among undergraduate occupational therapy students. An appropriate workload does not necessarily mean that the students invest less of themselves in their studying. Instead, time can be used in a different and more inspiring way. In line with Lizzio and co-workers [20], courses which are “less packed” may provide the students with a greater possibility to develop generic skills. When the workload is not excessive, it allows the student to use analytic, problem-solving and interactive learning processes, connected to the preferred deep study approach.

Similarly, students with lower scores on “unclear goals and standards” had higher scores on the surface approach to studying. Perceiving goals and standards of the study program to be unclear can make it difficult to see the purpose of the course they are attending, and a lack of purpose may indeed be considered an aspect of the surface approach to studying [38]. Students tend to adopt a surface approach to studying as a ‘default option’, when they are uncertain what the academic environment requires of them [39].

A preferred learning environment is one in which students know what will be expected from them, and therefore know what they need to do to manage these expectations. Diseth [18] underscored the importance of clarifying the goals and standards in the education program, thus enabling the students to cope with the learning material so that they do not experience overload. Tuononen and co-workers [22] emphasized the need for students to understand the importance and relevance that generic skills have for their future work. Therefore, clarifying the goals and standards concerned with generic skills acquisition may be especially important.

### Sociodemographic covariates to study approaches

In line with previous findings [37], this study revealed that female students and students who spend more time on independent study were more likely to use strategic approaches to learning. Other studies have also noted that female students appear to be more inclined than male students to use a deep study approach, and less inclined to use a surface approach [12, 40, 41]. Although the current study found gender differences only to be concerned with the strategic approach, a broader interpretation taking into account the results of the above-cited studies may indicate that female students are more inclined to use productive study approaches than men.

The detected relationship between higher age and deep approach to studying is also in line with previous research [12, 34, 42]. Being older naturally increases the possibility of having prior higher education, and the latter was also directly associated with higher deep approach scores. Both age and academic experience may add to the students maturity, as suggested from prior research – students who had previous experience from higher education had higher average exam grades, compared to their counterparts without similar experience [43].

Students whose current line of study was not their first choice had higher scores on surface approaches to studying. Students who originally wanted to study something other than occupational therapy may have experienced lower motivation and may have had less knowledge about the occupational therapy education program, compared to their counterparts. For those reasons, it may have been more difficult to get started with the learning activities and also to navigate in the curriculum, possibly leading to surface approach behaviors.

### Study strengths and limitations

Several strengths and weaknesses related to the study should be noted. The sample size was appropriate for the analysis, by well exceeding a recommended ratio of 15 participants per independent variable [44]. Participants were recruited from all six Norwegian education institutions offering occupational therapy education, and all of these aspects add to the validity of the study results. However, group sizes and response rates were different between the study sites, suggesting that the results are somewhat ‘weighted’ with students from some education institutions having more impact on the results than others. The cross-sectional study design (i.e., assessments performed at one point in time) precludes us from making causal interpretations about the direction of the detected associations. Associations between perceptions of the learning environment and individual study approaches may also be cyclical and self-strengthening in their nature.

In particular, the findings concerned with generic skills require one additional comment. Some researchers consider this scale to be more appropriately used when measuring the outcome from a learning process, rather than measuring an aspect of the learning environment [29]. According to this view, scores on the generic skills scale express the students’ evaluation (not their experience) of how the study program contributes to their generic learning outcomes. However, we would rather suggest a connection between the two views on generic skills. Perceiving the course to contribute to generic skills while taking the course (i.e., generic skills as learning environment factor) may indeed translate into better actual generic skills at the conclusion of the course (i.e., generic skills as learning outcome).

The study is based on self-report data only. While alternatives to self-report data pertaining to affective and attitudinal states are sparse, future studies may supplement the data collection by adding objective measures (e.g., related to workload) that can be compared to the subjective measures. Several studies have suggested that subjective perceptions of workload are not good measures of actual workload, the latter being a complex function of a range of factors [18, 45].

## Conclusion

The study aimed to examine associations between occupational therapy students’ perceptions of the learning environment and their approaches to studying, while adjusting for sociodemographic factors. We found that students who evaluated the study program as adding to their generic skills were more inclined to use deep and strategic study approaches, and less inclined to use a surface approach to studying, compared to their counterparts. Further, students who were more inclined to perceive the study program to be unclear about the goals and standards, and to pose an excessive workload, were more inclined to use the surface approach. Based on this study, we generally conclude that appropriately addressing the noted learning environment factors in occupational therapy education may assist students in adopting a productive approach to studying.

## Data Availability

The datasets generated during and/or analysed during the current study are not publicly available due to the still ongoing research process, but are available from the corresponding author on reasonable request.

## Abbreviations

ASSIST: Approaches and study skills inventory for students
CEQ: Course experience questionnaire
M: Mean
SD: Standard deviation
SOLO: Structure of Observed Learning Outcomes
